# Anthropometric Profile, Overweight/Obesity Prevalence, and Socioeconomic Impact in Moroccan Children Aged 6–12 Years Old with Autism Spectrum Disorder

**DOI:** 10.3390/ijerph21060672

**Published:** 2024-05-24

**Authors:** Rachid Touali, Maxime Allisse, Jamal Zerouaoui, El Mahjoub Chakir, Dominic Gagnon, Hung Tien Bui, Mario Leone

**Affiliations:** 1 Faculty of Sciences, Ibn Tofail University, Kenitra 14000, Morocco; rachid.touali1@uit.ac.ma (R.T.); jamal.zerouaoui@uit.ac.ma (J.Z.); elmahjoub.chakir@uit.ac.ma (E.M.C.); 2 Faculty of Physical Activity Sciences, University of Sherbrooke, Sherbrooke, QC J1H 5N4, Canada; maxime.allisse@usherbrooke.ca; 3 Jonquière Médic, Jonquière, QC G7X 7W6, Canada; dominic.gagnon.med@ssss.gouv.qc.ca; 4 Faculty of Medicine and Health Sciences, University of Sherbrooke, 3001 12 Ave N Immeuble X1, Sherbrooke, QC J1H 5N4, Canada; hung.tien.bui@usherbrooke.ca

**Keywords:** cardiometabolic risk, composite risk score, ASD children, central obesity, general obesity

## Abstract

Background: In addition to the inherent challenges of their condition, children with autism spectrum disorder (ASD) are also susceptible to the global obesity epidemic. However, concerning the prevalence of obesity within the Moroccan ASD pediatric population, data remain scarce. Methods: A total of 258 children (boys = 195) aged 6 to 12 years old (mean = 9.4 ± 1.4) diagnosed with ASD participated in this study. Besides the body mass and height, four significant anthropometric markers for assessing obesity were examined: body mass index (BMI), body surface area (BSA), waist circumference (WC), and waist-to-height ratio (WHtR). Each anthropometric marker was categorized into one of three cardiometabolic risk levels based on the Z-scores and their corresponding percentiles. The distribution was as follows: low risk (≤84th percentile), high risk (85th–94th percentile), and very high risk (≥95th percentile). Subsequently, a multiple regression analysis was employed to develop an algorithm that generates a composite risk score. This score incorporates all the anthropometric variables simultaneously, while also weighting their individual contributions to the cardiometabolic risk. Results: Children with ASD exhibit an anthropometric profile that markedly increases their susceptibility to cardiometabolic issues. While roughly 11% of the general Moroccan child population is overweight or obese, this figure soars to nearly 60% among children with ASD when considering the central adiposity markers. Furthermore, children from middle-class socioeconomic backgrounds display a more than threefold greater risk of developing overweight or obesity compared to their counterparts from lower socioeconomic backgrounds. Conclusions: This study has, for the first time, provided an up-to-date overview of the cardiometabolic risk in Moroccan children with ASD using traditional anthropometric measurements. The primary risk factor is clearly linked to central (abdominal) adiposity, which is recognized as the most deleterious. This study highlights the need to include general and central obesity markers. This study underscores the importance of incorporating both general and central adiposity markers for a more comprehensive assessment, and it emphasizes the need for closer monitoring within this high-risk population.

## 1. Introduction

### 1.1. Autism Spectrum Disorder (ASD)

Autism spectrum disorder (ASD) is a neurodevelopmental condition characterized by persistent difficulties in social interaction, communication, and stereotyped behaviors with restricted interests [1]. It manifests in early childhood and persists throughout life, with a wide range of symptoms and severity levels. Globally, the prevalence of ASD is estimated at approximately 1 in 100 individuals [2]. However, the presence of significant disparities across regions highlights the need for a nuanced understanding of the specific profile of affected populations. Notably, data concerning children with ASD remain scarce in emerging countries [3].

### 1.2. The Complex Link between ASD and Obesity

In addition to the challenges faced by children with ASD, they, like the broader typical pediatric population, are also impacted by the overweight and obesity epidemic. In fact, the prevalence of obesity in children with ASD tends to surpass that in neurotypical children, although quite notable variations exist across studies [4,5,6,7,8,9].

Several factors may contribute to this increased prevalence. Firstly, children diagnosed with ASD often experience motor skills challenges that can limit their participation in sports and other physical activities [10,11,12]. Additionally, selective eating preferences and sensory difficulties related to food are common in children with ASD [12,13,14,15]. Furthermore, specific classes of psychotropic medication are potentially implicated in obesity development among these children [7,16]. The consequences of obesity in children with ASD are manifold. Children with ASD face a multitude of consequences from obesity, with one of the most significant being the premature development of metabolic syndrome. This complex condition is characterized by a set of risk factors that dramatically increase susceptibility to cardiovascular diseases, type 2 diabetes, high blood pressure, high cholesterol, and other metabolic disorders [3,4,9]. Moreover, obesity can also exacerbate the symptoms associated with ASD. Physical health issues can lead to a deterioration in the overall quality of life for the child and increase the already present social and behavioral difficulties [17,18,19]. While research on obesity, its causes, and its effects in ASD populations has grown significantly in recent years, there is significant disparity in the research interest in this topic between countries.

### 1.3. Research Gap in ASD and Obesity in Morocco

Particularly in emerging countries such as Morocco, data on ASD children remain scarce [3,10,20,21,22]. While there are available data on ASD obesity among young Moroccan children under the age of 12 residing abroad [23,24], the prevalence of ASD within Morocco itself remains elusive. The lack of epidemiological information on the autism prevalence accentuates the need for comprehensive research within this particular population.

There are multiple reasons for this lack of specific data on autism in Africa in general and in Morocco in particular. Firstly, there is a gap in awareness and recognition of autism as a neurological disorder within Moroccan society, perhaps leading to its under diagnosis. Furthermore, medical resources and specialized facilities for the diagnosis and treatment of autism are limited in the country [18,25]. This scarcity contributes to the challenges in gathering comprehensive data on the prevalence of autism among Moroccan children. However, it is important to note that efforts are underway to improve the situation. Organizations, healthcare professionals, and support groups are working together to increase awareness, while initiatives aimed at improving access to diagnostic and intervention services are also being developed.

### 1.4. Rational of the Study

Collecting precise data on the prevalence of autism, particularly regarding overweight and obesity among Moroccan children, is essential to inform health policies, allocate resources effectively, and plan services accordingly. It will also help to better understand the specific needs of this population and provide adequate support to families and autistic children.

### 1.5. Objectives

This study aimed to achieve three objectives: (1) to update the anthropometric profile of autistic Moroccan children aged 6 to 12 years old compared to their neurotypical peers; (2) to evaluate the prevalence of overweight and obesity in this population; and (3) to examine the impact of socioeconomic status (SES) on the prevalence of overweight and obesity among these children with ASD.

## 2. Materials and Methods

### 2.1. Design

This study is a descriptive comparative study with a cross-sectional design conducted on a sample of Moroccan children with ASD aged 6–12 years old.

### 2.2. Participants

A total of 258 children (boys = 195) aged 6 to 12 years old (mean = 9.4 ± 1.4) diagnosed with ASD participated in this study. Data collection was undertaken between March and May of 2023. Participants were recruited from three non-profit organizations. These organizations are situated in two primary regions: Tetouan, with 153 children, and Rabat/Salé, with 105 children. Both regions are classified as urban areas. Each child underwent assessment by a psychiatrist associated with their respective organization. Participants were categorized based on the severity of their symptoms according to the Diagnostic and Statistical Manual of Mental Disorders (DSM-5) into three groups. (1) Mild ASD (N = 33): Individuals in this group likely experience challenges in social communication and interaction, with some difficulties interpreting nonverbal cues or initiating conversations. They may also exhibit restricted or repetitive behaviors, but these are likely to be less pervasive and cause less functional impairment in daily life. (2) Moderate ASD (N = 168): This group likely experiences more significant difficulties in social communication and interaction. They may struggle to understand and respond to social cues, have limited or inflexible conversation skills, and find it challenging to develop and maintain friendships. Additionally, restricted or repetitive behaviors may be more pronounced and interfere with some aspects of daily life. (3) Severe ASD (N = 57): This group experiences the most severe symptoms of ASD. Social communication and interaction are likely to be extremely challenging, with individuals potentially being nonverbal or relying heavily on alternative communication methods. Restricted or repetitive behaviors are likely to be very limiting and significantly impact daily routines and functioning across multiple environments. SES was determined based on family income, in accordance with guidelines from the High Commission for Planning of Morocco: low income < MAD 3000 per month, and average income ≥ MAD 3000 per month.

### 2.3. Anthropometric Measures

Anthropometric measurements, including body mass (BM), body height (BH), body mass index (BMI) and body surface area (BSA), were collected in the classrooms of the three associations by a member of our team (RT), assisted by the specialized educators’ team. BM was recorded to the nearest 0.5 kg using a Seca scale model 760 (Hamburg, Germany). BH was measured using a Seca stadiometer model 214 (Hamburg, Germany) to the nearest 0.1 cm. Waist circumference (WC) was assessed using measuring tape to the nearest 0.1 cm in accordance with the guidelines provided by the WHO. All the selected measurements adhered to standardized procedures as recommended by Lohman et al. [26].

BMI was calculated using the formula $\frac{BM\;\left(kg\right)}{BH^{2}\left(m\right)}$. Following the classification proposed by Cole et al. [27] and endorsed by the World Health Organization (WHO), each child was categorized as typical, overweight, or obese. This classification leverages data from multiple countries to establish an international standard for BMI assessment in children. To achieve this, the Z-scores were computed based on individual BMI values, along with age (divided into half-year increments) and sex, using international reference data. Children whose BMI falls below the 84th percentile are considered to have a typical BMI (Z-score < 0.994). Those between the 85th and 94th percentiles are classified as overweight (Z-score = 0.996 to 1.555). Finally, children with a BMI exceeding the 95th percentile are categorized as obese (Z-score > 1.644).

BSA was estimated using the method suggested by Mosteller [28], which read as follow:

$$\mathrm{BSA}\;(m^{2})=\sqrt{\frac{BH\;\left(cm\right)\times BM\;(kg)}{3600}}$$

Finally, the waist-to-height ratio (WHtR) was calculated using the following formula:

$$\mathrm{WHtR}\;(\mathrm{ratio})=\frac{WC\;\left(cm\right)}{BH\;\left(cm\right)}$$

### 2.4. Statistical Analysis

Descriptive statistics are depicted as the mean ± standard deviation (SD), along with the 95% confidence interval (CI). The Cohen’s d effect sizes were calculated to assess the magnitude of differences between groups. The Shapiro–Wilk test was used to assess the normality of each variable’s distribution. For variables with non-normal distributions, a Box–Cox transformation was applied to normalize the data [29]. Group comparisons were conducted using unpaired *t*-tests and/or Pearson’s χ as required. However, since no significant differences were observed among the three categories of symptom severity, these data are not presented. A stepwise multiple regression analysis was conducted to build an equation using anthropometric markers as the independent variables to estimate an individual’s overall health risk (composite risk score). The Bland–Altman analysis was used to compare the measured and predicted composite risk score (CRS) values. All the statistical analyses were carried out using the IBM-SPSS software version 24.

As suggested by numerous studies, the cutoff point for assessing the cardiometabolic risk based on anthropometric variables typically falls at the 85th percentile for overweight and the 95th percentile for obesity [30,31,32,33]. Given the limited data available in Morocco for the age group concerned, we opted for this classification for the sake of simplicity:

BMI, BSA, WC and WHtR Low risk < 85th percentile High risk = 85th–95th percentile Very high risk > 95th percentile

As recently shown, simultaneous analysis of multiple anthropometric variables offers a more precise assessment of the cardiometabolic risk [34]. This approach provides a more complete picture of an individual’s metabolic profile, surpassing the limitations of evaluating each variable in isolation. The CRS is derived by aggregating the risks associated with each of four key anthropometric markers: BMI, BSA, WC, and WHtR. A low risk is assigned a value of 1, a moderate risk is 2, and a very high risk is 3. Consequently, the CRS ranges from 4, indicating a minimal risk, to 12, representing the maximum risk level (∑BMI + BSA + WC + WHtR). Based on this scale, the final CRS is calculated as follows:

Low risk ≤ 5 High risk = 6–9 Very high risk ≥ 10

## 3. Results

Table 1 presents descriptive statistics of the children with ASD in terms of the BM, BH, BMI, BSA, WC and WHtR stratified by age and sex. Notably, the male-to-female ratio for ASD in this sample is approximately 3:1, which aligns with established prevalence patterns for this disorder [25].

Data analysis reveals a noticeable distinction between the ASD boys and girls of the same age group, as girls demonstrate significantly higher BM and BMI compared to boys Regarding the WHtR, although no statistically significant difference is observed between the sexes, the effect size indicates a moderate effect, suggesting a potential clinical relevance.

Table 2 provides an in-depth comparison of anthropometric markers between neurotypical and ASD children. Data for the BM, BH, and BMI in the neurotypical group were obtained from the Ministry of National Education, Professional Training, Higher Education, and Scientific Research of Morocco [35]. Because there was a lack of data specifically for Moroccan children regarding the WC and WHtR, the comparison group was sourced from a Tunisian study [36]. This decision was guided by the acknowledged sociodemographic and cultural similarities between these two populations.

While there are no significant differences in the BH, boys with ASD in this study tend to have slightly higher statistically significant BM, BMI, and BSA compared to neurotypical children. However, the effect sizes for these measures are small, suggesting minimal clinical differences. The WC and WHtR measurements also reveal statistically significant disparities between the groups. This observation is further strengthened by the clinically relevant effect sizes. In the girls with ASD, all the anthropometric markers exhibit strong statistically significant differences and are accompanied by high effect sizes.

**Figure 1:**
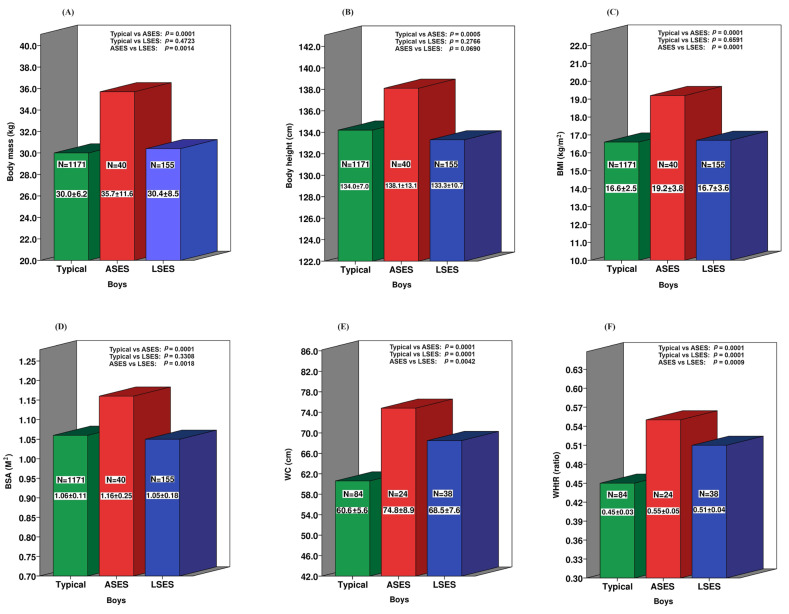
Socioeconomic disparities in the anthropometric measurements between ASD and neurotypical boys. (**A**) Body mass; (**B**) Body height; (**C**) Body mass index; (**D**) Body surface area; (**E**) Waist circumference; (**F**) Waist-to-height ratio.

A more nuanced examination of the ASD group, stratified by SES, unveils a distinct pattern. As depicted in Figure 1, ASD boys with low SES (LSES) display a morphological profile comparable to that of neurotypical children in terms of the BM, BH, and general obesity as indicated by the BMI and BSA, with no statistically significant difference observed.

Conversely, ASD boys hailing from average socioeconomic (ASES) backgrounds manifest statistically significant disparities across all the morphological markers, encompassing both central and general obesity. This discernible contrast persists even upon comparison with their counterparts from LSES backgrounds.

**Figure 2:**
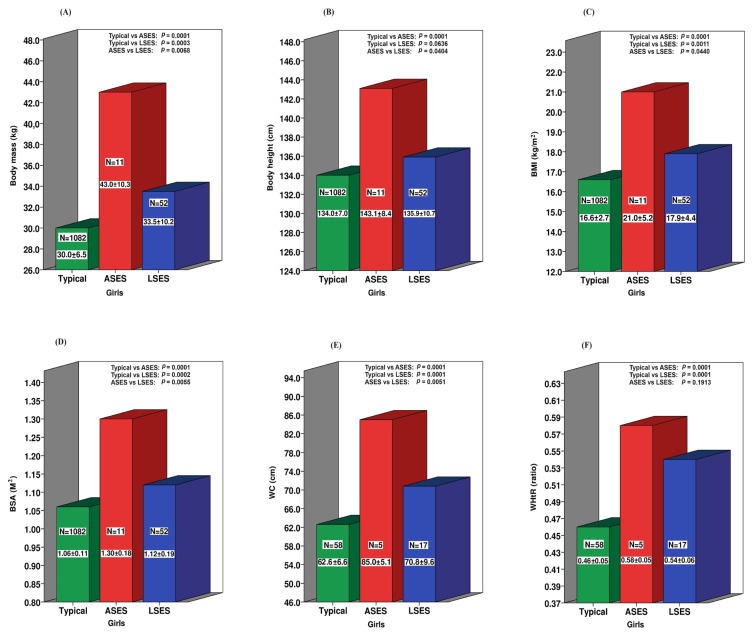
Socioeconomic disparities in the anthropometric measurements between ASD and neurotypical girls. (**A**) Body mass; (**B**) Body height; (**C**) Body mass index; (**D**) Body surface area; (**E**) Waist circumference; (**F**) Waist-to-height ratio.

Figure 2 highlights that among the girls, all the anthropometric indicators are significantly higher in the ASES group compared to neurotypical girls. In contrast to boys, the LSES girls exhibited higher values for all the anthropometric markers except for the BM, which borders statistical significance (*p* = 0.0636). This pattern is also evident when comparing the two SESs, with the ASES girls having an increased risk of cardiometabolic issues, even though the WHtR value does not reach statistical significance (*p* = 0.1913). However, these values should be interpreted cautiously due to the small sample sizes for central obesity variables such as the WC and WHtR.

Table 3 provides strong evidence that SES is a significant risk factor for cardiometabolic conditions in children with ASD. This table focuses on the key markers commonly used to assess the cardiometabolic risk linked to childhood obesity. The data reveal a significantly higher prevalence (odds ratio) of these markers in children with ASD from average SES (ASES) backgrounds compared their counterparts from lower SES (LSES) backgrounds. For instance, children with ASD from ASES backgrounds have a 3.2-fold increased risk of having a BMI exceeding the 85th percentile, indicating a greater predisposition to overweight or obesity. The CRS serves as a comprehensive indicator of the cardiometabolic risk by simultaneously considering all the essential variables. By incorporating both central (abdominal) and general adiposity, the CRS provides a more nuanced assessment of the overall cardiometabolic risk.

Table 4 demonstrates the strong performance by the model. The measured and predicted average CRS scores are very close, indicating the model’s effectiveness in overall prediction. Additionally, the similar variability observed in both sets of scores suggests a high degree of concordance between the measured and predicted data. The high R^2^ value of 0.854 further bolsters this conclusion, signifying that the model explains a substantial 85.4% of the variability in the measured CRS scores. The stepwise multiple regression analysis successfully identified three key variables for accurate CRS prediction: WC, BMI, and age. These variables are particularly relevant as the WC and BMI represent the two most commonly used markers of obesity (abdominal and general, respectively), while age captures a potential risk factor for metabolic changes. Furthermore, the absence of significant multicollinearity, as evidenced by the VIF range, indicates that the independent variables (WC, BMI, age) are not redundant and provide distinct contributions to the model. Finally, the model evaluation metrics (R^2^, RMSE, MAE, SEE, VIF) collectively suggest that the model achieves an adequate level of accuracy in its predictions.

**Figure 3:**
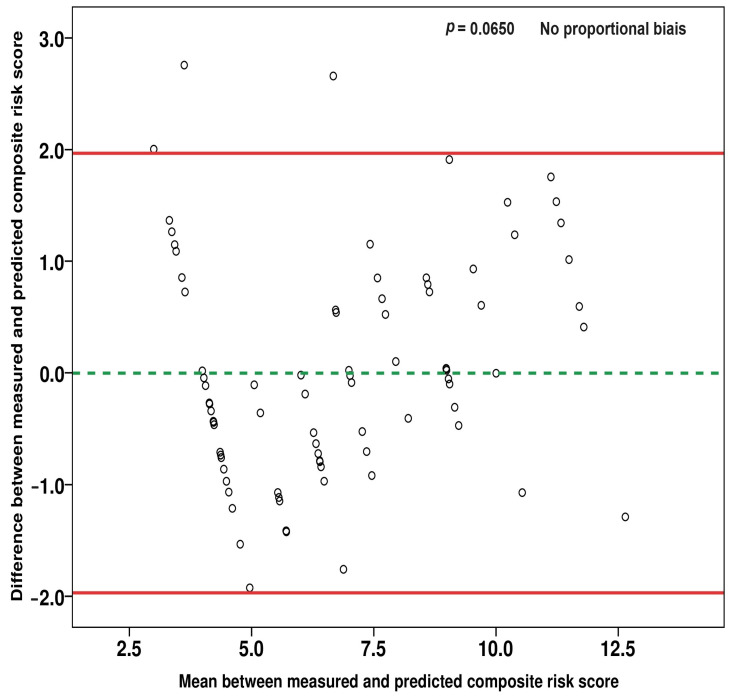
Bland–Altman plot comparing the measured versus the predicted values of the CRS. The two red lines represent the confidence interval (CI95%) and the green dashed line represents the mean of the differences between the two compared methods of measurements.

Figure 3 presents the Bland–Altman plot providing a detailed visualization of the agreement between the two datasets. Essentially, this graph confirms the accuracy and reliability of our model in predicting the CRS. The absence of proportional bias and the low variability between the measured and predicted values adds confidence concerning the model’s ability to provide accurate CRS estimates.

Further examination of the data presented in Table 5 underscores the significant prevalence of overweight and obesity among children with ASD. Specifically, the individual assessment of two anthropometric markers associated with general obesity, BMI and BSA, indicates that nearly one-third (32.5%) of these children exhibit a high (overweight) or very high risk (obesity) of developing cardiometabolic complications. This risk becomes even more pronounced when considering the two markers associated with central obesity, WC and WHtR. In this scenario, the proportion of children at high or very high risk climbs to nearly 60%, underscoring the substantial prevalence of abdominal obesity and the heightened risk to cardiovascular health in children with ASD. When evaluating all four markers concurrently, we obtain a score of 58.4%, which likely represents the most accurate assessment of risk. Individual assessment of the CRS can be simplified using the three-factor regression equation.

Table 6 presents the prevalence of overweight and obesity in school-aged children, categorized by age (two distinct groups) and sex. This dichotomous analysis allows for a more precise evaluation of how these two factors influence the potential for developing future cardiometabolic risks. The data reveal a general trend of an increasing risk of overweight and obesity with age, observed in both boys and girls. However, this risk appears to be significantly higher among girls overall, particularly in the younger age group (6–9 years old).

## 4. Discussion

Addressing childhood autism in emerging countries presents a multifaceted challenge due to the complex interplay of cultural, socioeconomic, and educational barriers. Firstly, a significant lack of awareness and understanding of autism permeates many communities. Persistent stereotypes and prejudices can lead to fear, stigmatization, and social rejection of children with ASD and their families. The limited medical and educational resources available in developing countries often hinder diagnosis of and intervention for autism. This is further complicated by the specific challenges of diagnosing ASD [25]. Families may face significant difficulties accessing qualified healthcare professionals and specialized services, which can significantly delay diagnosis and crucial treatment [19]. The financial burden associated with therapies and interventions compounds this problem, creating a near-insurmountable obstacle for families struggling economically. Having established the general challenges of addressing ASD in developing countries, this study focuses on the specific issue of obesity among Moroccan children with ASD.

### 4.1. Obesity in Moroccan Children with ASD

Beyond the problems inherent to their condition, children with ASD are also struggling with the obesity epidemic, which is currently affecting all societies. Understanding this phenomenon within the Moroccan population is notably intricate due to the initial absence of information, even in a general sense, concerning ASD cases in this community. Specifically, there is a lack of official national data regarding the prevalence of this condition, particularly among children aged 12 and under. Consequently, estimating cases of overweight and obesity specifically within this age cohort poses a substantial challenge. While preliminary, the data outlined in this study represent a significant leap forward in our understanding of obesity within the Moroccan children with ASD population. This study offers the first glimpse into the prevalence of overweight and obesity in this particular demographic, addressing a critical knowledge gap concerning a crucial public health issue. Indeed, these initial findings provide valuable insights into the reality of obesity among Moroccan children with ASD. They identify a population at a heightened risk of weight-related concerns, paving the way for further investigation and the development of targeted interventions aimed at preventing and managing obesity within this community.

### 4.2. A Crucial First Step: Unveiling the Prevalence of Obesity among Moroccan ASD Children

This study illuminated previously obscure characteristics of Moroccan children with ASD. Notably, the severity of the symptoms did not impact their body composition. A recent study supports this finding, though some controversy surrounding this topic persists [37]. The analysis also reveals a distinct anthropometric profile in Moroccan children with ASD compared to their neurotypical counterparts for all the markers studied. Among boys, only the BH did not significantly differ between the two groups. This finding highlights that, for a given height, markers associated with central obesity such as the WC and WHtR exhibit markedly higher values in boys with ASD. These substantial disparities (Cohen’s effect sizes of 1.67 and 1.40 respectively) suggest a higher risk of developing a cardiometabolic condition in the long term. The pattern is even more pronounced among girls, with all the analyzed anthropometric markers displaying highly significant differences, accompanied by large effect sizes. However, the significant difference observed in the BH for girls with ASD might suggest earlier physical maturity compared to their neurotypical counterparts. However, our study design did not directly assess menarche, the onset of menstruation, in the participating girls. Notably, only 6 out of the 63 girls fell within the age range of 11.5 to 12.5 years, typically associated with menarche. With only a small sample of girls within the typical menarche age range, the potential influence of hormonal changes on body composition is unlikely to have played a significant role in our findings. However, due to the limited sample size, primarily consisting of girls in the 10–12-year age group, these results warrant cautious interpretation. In a study involving adolescent girls with ASD, menarche would be a crucial factor to consider. Our findings rather suggest a potential divergence in the body fat distribution among children with ASD, possibly increasing their susceptibility to developing cardiometabolic issues. This pattern suggests an elevated risk of cardiometabolic complications, supported by both the overall adiposity (BMI and BSA) and the central adiposity measures (WC and WHtR).

Given the multitude of factors that can influence the observed disparities between children with ASD and neurotypical children, a preventive approach appears to be the most promising strategy. This study further emphasizes the importance of developing early intervention strategies to curb excessive weight gain, especially in young girls with ASD. Such targeted approaches could significantly reduce their risk of developing cardiometabolic health problems later in life. It is important to emphasize that our use of various anthropometric measurements (BMI, BSA, WC, WHtR) provides a more comprehensive assessment of the body composition status of children. Each measurement offers valuable and complementary insights, enhancing the precision of identifying children at risk.

#### Cardiometabolic Risk Profile across Socioeconomic Backgrounds

This study reveals a comparable cardiometabolic risk profile for the BH, BM and general obesity markers in boys with a lower SES compared to age-matched neurotypical Moroccan children. Nonetheless, a significant contrast emerges in the central obesity markers, indicating a higher predisposition toward developing an excess of abdominal adiposity in boys with ASD. Furthermore, boys from average SES (ASES) backgrounds present an increased cardiometabolic risk for all the anthropometric markers compared to their neurotypical counterparts. This disparity is also observed between ASES and LSES backgrounds, indicating a more unfavorable profile among children with ASD with a higher SES. It is thus crucial to highlight the increased risk of cardiometabolic complications in these boys, given the elevated values of the central adiposity markers as indicated by the WC and WHtR. These findings underscore the pivotal influence of SES in predisposing boys with ASD to cardiometabolic disorders, highlighting the complex interplay between socioeconomic factors and health outcomes in this population.

Girls with ASD appear to be even more at risk, as even those from lower SES backgrounds exhibit significant differences in all the anthropometric markers compared to neurotypical girls. Therefore, although children of both sexes with ASD appear to be at an increased risk of developing cardiometabolic issues, girls appear to be particularly vulnerable. In fact, children with ASD from middle-income households are over three times more likely to have anthropometric markers exceeding the 85th percentile, suggesting a higher risk of overweight or obesity.

At first glance, these findings may appear counterintuitive, as in high-income countries, children from lower SES backgrounds are typically regarded as being the most affected by the obesity pandemic [37]. It is worth noting that similar findings have also been reported in other developing countries [38]. However, there are several factors that may contribute to this trend. In developing countries, children from low-income households may encounter restricted access to high-calorie and high-fat foods, which may contribute to a diet that is less likely to cause obesity. Moreover, lifestyles in these regions often entail greater physical activity, such as walking to school or engaging in household chores, which can mitigate BM gain. Conversely, children from higher-income families in developing countries may have easier access to these very same calorie-dense, processed foods with limited nutritional value. Coupled with potentially more sedentary lifestyles characterized by frequent car use and extensive screen time, these factors may contribute to a higher prevalence of obesity among this demographic.

While the available data strongly suggest that these factors play a role, it remains unclear whether their influence is equal within families of children with ASD. For instance, physical activity demonstrably affects body composition, but no significant difference in activity levels has been established between LSES and ASES families raising children with ASD. This suggests a more nuanced picture, likely involving a complex interplay of all the aforementioned factors.

### 4.3. Beyond Individual Measures: CRS for Cardiometabolic Risk Assessment

Despite its advantages in terms of the simplicity and strong correlation with various health markers, the BMI faces criticism regarding its accuracy in measuring overweight or obesity, particularly in young people [34,39,40,41]. The primary concern lies in the BMI’s role as an overall measure, as it fails to distinguish between adipose tissue and lean mass, offering no insights into body fat distribution. Accordingly, including complementary reliable markers is crucial to corroborate BMI-derived trends. Thus, by capturing distinct aspects of obesity—general fat distribution (BMI) and central adiposity (WC)—their combined use could likely refine our understanding of the cardiometabolic risk.

As demonstrated in this study, evaluating different obesity markers individually offers initial insight into the cardiometabolic risk in children with ASD. Nevertheless, significant variations emerge depending on whether general or central obesity markers are considered (~32% versus ~58%, respectively). Moroccan studies providing BMI values for children in the general population are rare. However, in 2013, Sebbani et al. [42] reported a combined prevalence of overweight and obesity of 11% based on BMI values according to the WHO criteria. Even when compared with the IOTF values as reported in this study (17.6%), these values remain significantly lower than what we have observed in children with ASD. As for the WC, no standardized value relating to the Moroccan child population has been found, making any comparison highly uncertain.

#### Why Use the CRS?

The composite risk score (CRS) offers the advantage of summarizing the cardiometabolic risk by simultaneously analyzing the anthropometric markers. Stepwise multiple regression analysis yielded an equation for assessing the cardiometabolic risk that considers the interaction between all the model variables. The final algorithm identified two anthropometric markers (BMI and WC), including age, as adequate for a highly accurate assessment of the metabolic complication risk in children with ASD. This method simplifies the result interpretation for clinicians by incorporating both general and central obesity markers while modulating their individual contributions.

### 4.4. Limitations and Strengths

This study presents several limitations. First, the analysis utilizes anthropometric markers derived from field measurements. While convenient, these measurements may not always attain the precision necessary for drawing robust inferences compared to direct measures. Then, the lack of data from rural areas restricts the generalizability of our findings. The use of Tunisian data to compare the WC and WHtR is also a limitation. Additionally, the cross-sectional design of this study restricts our ability to draw causal conclusions. However, this study also possesses significant strengths. Notably, it is the first Moroccan study to assess the prevalence of overweight and obesity in children with ASD aged 6 to 12 years. Furthermore, the sample size of 258 children is sufficient to generate adequate statistical power. At the very least, these data provide an objective preliminary picture of the cardiometabolic risk in children with ASD. Finally, the proposed model could potentially be applied to the general Moroccan population, thus contributing to a more comprehensive characterization of this population.

## 5. Conclusions

This study has, for the first time, provided an up-to-date overview of the cardiometabolic risk in Moroccan children with ASD using traditional anthropometric measurements. Our results indicate that children with ASD from urban environments face an elevated risk of developing obesity-related cardiometabolic issues. Indeed, the prevalence of overweight and obesity within this population is significantly higher compared to neurotypical Moroccan children. This observation is particularly concerning for children with ASD from middle socioeconomic backgrounds. The primary risk factor is clearly linked to central (abdominal) adiposity, which is recognized as the most deleterious. However, the proposed model transcends this by incorporating and weighting the combined influences of both general and central obesity factors, leading to a more refined characterization of the risk. This straightforward and adaptable method holds promise for application across the broader Moroccan population.

## Figures and Tables

**Table 1:** Anthropometric characteristics of Moroccan autistic children aged 6–12 years old.

Variables	Boys (N = 195)	CI95%	Girls (N = 63)	CI95%	*p* Values	Cohen’s d Effect Size
Age (years)	9.4 ± 1.4	9.2–9.6	9.6 ± 1.4	9.2–9.9	0.285	0.14
BM (kg)	31.5 ± 9.4	30.2–32.9	35.2 ± 10.8	32.4–37.9	**0.011**	0.38
BH (cm)	134.2 ± 11.3	132.6–135.8	137.2 ± 10.6	134.5–139.8	0.073	0.27
BMI (kg·m^2^)	17.2 ± 3.8	16.7–17.8	18.5 ± 4.6	17.3–19.7	**0.031**	0.32
BSA (m^2^)	1.08 ± 0.20	1.05–1.10	1.15 ± 0.20	1.10–1.20	**0.013**	0.35
WC (cm) *	71.0 ± 8.6	68.8–73.2	74.1 ± 10.4	69.4–78.7	0.175	0.34
WHtR (ratio) *	0.52 ± 0.05	0.51–0.54	0.55 ± 0.06	0.52–0.57	0.100	0.57

BM = Body mass; BH = Body height; BMI = Body mass index; WC = Waist circumference; WHtR = Waist-to-height ratio; BSA = Body surface area; * WC and * WHtR N = 62 for boys and N = 22 for girls; CI95% = Confidence interval at 95%; *p* values **Bold** = Significant at *p* ≤ 0.05; Cohen’s d effect size: <0.20 = marginal; 0.20–0.49 = small; 0.50–0.79 moderate; ≥0.80 = large.

**Table 2:** Anthropometric comparisons of neurotypical and ASD Moroccan children aged 6–12 years.

**Variables**	**Neurotypical** **(N = 1171)**	**All ASD** **(N = 195)**	***p* Values**	**Cohen’s d** **Effect Size**
**Boys**				
Body Mass (kg)	30.0 ± 6.2	31.5 ± 9.4	**0.0041**	0.22
Body Height (cm)	134.0 ± 7.0	134.2 ± 11.3	0.7390	0.00
BMI (kg/m^2^)	16.6 ± 2.5	17.2 ± 3.8	**0.0045**	0.22
BSA (m^2^)	1.06 ± 0.11	1.08 ± 0.20	**0.0415**	0.16
* WC (cm)	60.0 ± 6.2	71.0 ± 8.6	**0.0001**	1.67
* WHtR (Ratio)	0.45 ± 0.05	0.52 ± 0.05	**0.0001**	1.40
**Variables**	**Neurotypical** **(N = 1082)**	**All ASD** **(N = 63)**	***p* Values**	**Cohen’s d ** **Effect Size**
**Girls**				
Body Mass (kg)	30.0 ± 6.5	35.2 ± 10.8	**0.0001**	0.76
Body Height (cm)	134.0 ± 7.0	137.2 ± 10.6	**0.0007**	0.46
BMI (kg/m^2^)	16.6 ± 2.7	18.5 ± 4.6	**0.0001**	0.67
BSA (m^2^)	1.06 ± 0.11	1.15 ± 0.20	**0.0001**	0.77
* WC (cm)	60.2 ± 6.9	74.1 ± 10.4	**0.0001**	1.95
* WHtR (Ratio)	0.46 ± 0.05	0.55 ± 0.06	**0.0001**	1.78

ASD = Autism spectrum disorder; BMI = Body mass index; BSA = Body surface area; WC = Waist circumference; WHtR = Waist-to-height ratio; p values **Bold** = Significant at *p* ≤ 0.05; Cohen’s d effect size: <0.20 = marginal; 0.20–0.49 = small; 0.50–0.79 moderate; ≥0.80 = large; * WC and * WHtR N = 62 for ASD boys and N = 84 neurotypical, N = 22 for ASD girls and N = 58 neurotypical.

**Table 3:** Socioeconomic status (SES) as a predictor of the cardiometabolic risk based on anthropometric markers in children with ASD.

Outcome	Odds Ratio Average vs. Low SES	95% CI		*p* Values (Pearson χ)
		Lower	Upper	
BMI	3.20	1.71	6.02	**0.0001**
BSA	3.36	1.79	6.33	**0.0001**
WC	2.97	1.04	8.44	**0.0300**
WHtR	4.06	1.49	11.1	**0.0006**
CRS	3.26	1.20	8.87	**0.0210**

SES = Socioeconomic status; CI95% = Confidence interval at the level of 95%; BMI = Body mass index; BSA = Body surface area; WC = Waist circumference; WHtR = Waist-to-height ratio; CRS = Cumulative risk for BMI, BSA, WC, and WHtR based on their respective Z-scores; **Bold** = Significant at *p* ≤ 0.05.

**Table 4:** Measured vs. predicted composite risk score (CRS) and stepwise multiple regression equation.

	N	Mean ± (SD)	95%CI	SEM	*p* Value	R^2^
Measured composite risk score	84	6.75 ± 2.63	6.18–7.32	0.29	0.977	0.854
Predicted composite risk score	84	6.75 ± 2.43	6.23–7.28	0.27		
Regression equation for CRS	R^2^	RMSE	MAE	SEE	Mean VIF	
CRS = −5.02 + (WC × 0.197) + (BMI × 0.277) + (Age × −0.797)	0.854	1.00	0.80	1.00	2.7 Range: 1.2–3.5	

N = Number of participants; SD = Standard deviation; 95%CI = Confidence interval; SEM = Standard error of the mean; *p* = Significant at *p* ≤ 0.05; R2 = Coefficient of determination; RMSE = Root mean square error; MAE = Mean absolute error; SEE = Standard error of estimate; VIF = Variance inflation factor.

**Table 5:** Percentage of ASD children who present a health risk according to anthropometric markers.

Markers	Low Risk	High Risk	Very High Risk	High + Very High Risk
BMI	67.1%	24.0%	8.9%	32.9%
BSA	67.8%	13.2%	19.0%	32.2%
WC	35.7%	28.6%	35.7%	64.3%
WHtR	45.2%	32.1%	22.6%	54.7%
CRS	41.7%	41.7%	16.7%	58.4%

BM = Body mass; BH = Body height; BMI = Body mass index; WC = Waist circumference; WHtR = Waist-to-height ratio; BSA = Body surface area; CRS = Composite risk score.

**Table 6:** Prevalence of overweight and obesity in school-aged children by anthropometric measure, age group, and sex.

BOYS 6–9 years old					
	OW	Obese	Typical	Total	OW + Obese
BMI (N)	27	10	90	127	29.2%
%	21.3	7.9	70.8	100	
BSA (N)	21	24	82	127	34.4%
%	16.5	18.9	64.6	100	
WC (N)	14	13	18	45	60.0%
%	31.1	28.9	40.0	100	
WHtR (N)	16	7	22	45	51.2%
%	35.6	15.6	48.8	100	
CRS (N)	18	5	22	45	51.1%
%	40.0	11.1	48.9	100	
**BOYS 10–12 years old**					
BMI (N)	15	6	47	68	30.9%
%	22.1	8.8	69.1	100	
BSA (N)	8	6	54	68	20.6%
%	11.8	8.8	79.4	100	
WC (N)	6	6	5	17	70.6%
%	35.3	35.3	29.4	100	
WHtR	6	4	7	17	59.8%
%	35.3	23.5	41.2	100	
CRS (N)	8	4	5	17	70.6%
%	47.1	23.5	29.4	100	
**GIRLS 6–9 years old**					
BMI (N)	12	4	21	37	43.2%
%	32.4	10.8	56.8	100	
BSA (N)	3	16	18	37	51.3%
%	8.1	43.2	48.7	100	
WC (N)	3	7	4	14	71.4%
%	21.4	50.0	28.6	100	
WHtR	5	4	5	14	64.3%
%	35.7	28.6	35.7	100	
CRS (N)	7	3	4	14	71.4%
%	50.0	21.4	28.6	100	
**GIRLS 10–12 years old**					
BMI (N)	8	3	15	26	42.3%
%	30.8	11.5	57.7	100	
BSA (N)	2	3	21	26	19.2%
%	7.7	11.5	80.8	100	
WC (N)	1	4	3	8	62.5%
%	12.5	50.0	37.5	100	
WHtR	4	0	4	8	50.0%
%	50.0	0.0	50.0	100	
CRS (N)	2	2	4	8	50.0%
%	25.0	25.0	50.0	100	

BMI = Body mass index; BSA = Body surface area; WC = Waist circumference; WHtR = Waist-to-height ratio; N = Number of participants; OW = Overweight; CRS = Composite risk score.

## Data Availability

The original contributions presented in the study are included in the article. Further inquiries can be directed to the corresponding authors.

## References

[1] American Psychiatric Association (2013). Diagnostic and Statistical Manual of Mental Disorders (DSM-5).

[2] Broder-Fingert S., Brazauskas K., Lindgren K., Iannuzzi D., Van Cleave J. (2014). Prevalence of overweight and obesity in a large clinical sample of children with autism. Acad. Pediatr..

[3] Kittana M., Ahmadani A., Williams K.E., Attlee A. (2023). Nutritional Status and Feeding Behavior of Children with Autism Spectrum Disorder in the Middle East and North Africa Region: A Systematic Review. Nutrients.

[4] Hill A.P., Zuckerman K.E., Fombonne E. (2015). Obesity and Autism. Pediatrics.

[5] de Vinck-Baroody O., Shui A., Macklin E.A., Hyman S.L., Leventhal J.M., Weitzman C. (2015). Overweight and Obesity in a Sample of Children with Autism Spectrum Disorder. Acad. Pediatr..

[6] Garcia J.M., Odahowski C.L. (2023). An urban versus rural comparison of obesity between youth with and without autism spectrum disorder. Autism Res..

[7] Sammels O., Karjalainen L., Dahlgren J., Wentz E. (2022). Autism Spectrum Disorder and Obesity in Children: A Systematic Review and Meta-Analysis. Obes. Facts.

[8] Zulkifli M.N., Kadar M., Hamzaid N.H. (2022). Weight Status and Associated Risk Factors of Mealtime Behaviours among Children with Autism Spectrum Disorder. Children.

[9] Curtin C., Anderson S.E., Must A., Bandini L. (2010). The prevalence of obesity in children with autism: A secondary data analysis using nationally representative data from the National Survey of Children’s Health. BMC Pediatr..

[10] Zhong T., Liu H., Li Y., Qi J. (2022). Correlates of Physical Activity of Children and Adolescents with Autism Spectrum Disorder in Low- and Middle-Income Countries: A Systematic Review of Cross-Sectional Studies. Int. J. Environ. Res. Public Health.

[11] Toscano C.V.A., Carvalho H.M., Ferreira J.P. (2018). Exercise Effects for Children with Autism Spectrum Disorder: Metabolic Health, Autistic Traits, and Quality of Life. Percept. Mot. Skills.

[12] Bandini L.G., Anderson S.E., Curtin C., Cermak S., Evans E.W., Scampini R., Maslin M., Must A. (2010). Food selectivity in children with autism spectrum disorders and typically developing children. J. Pediatr..

[13] Harris H.A., Bowling A., Santos S., Greaves-Lord K., Jansen P.W. (2022). Child ADHD and autistic traits, eating behaviours and weight: A population-based study. Pediatr. Obes..

[14] Kozak A., Czepczor-Bernat K., Modrzejewska J., Modrzejewska A., Matusik E., Matusik P. (2023). Avoidant/Restrictive Food Disorder (ARFID), Food Neophobia, Other Eating-Related Behaviours and Feeding Practices among Children with Autism Spectrum Disorder and in Non-Clinical Sample: A Preliminary Study. Int. J. Environ. Res. Public Health.

[15] Matheson B.E., Douglas J.M. (2017). Overweight and Obesity in Children with Autism Spectrum Disorder (ASD): A Critical Review Investigating the Etiology, Development, and Maintenance of this Relationship. Rev. J. Autism Dev. Disord..

[16] Dhaliwal K.K., Orsso C.E., Richard C., Haqq A.M., Zwaigenbaum L. (2019). Risk Factors for Unhealthy Weight Gain and Obesity among Children with Autism Spectrum Disorder. Int. J. Mol. Sci..

[17] Granich J., Lin A., Hunt A., Wray J., Dass A., Whitehouse A.J. (2016). Obesity and associated factors in youth with an autism spectrum disorder. Autism.

[18] Kantawala B., Abu-Bakr A., Kasini B., Ndayambaje M., Ian Soh S., Nazir A., Wojtara M., Uwishema O. (2023). Exploring the landscape of autism in Africa: Challenges in diagnosis, support, and resources—A short communication. Ann. Med. Surg..

[19] Ruparelia K., Abubakar A., Badoe E., Bakare M., Visser K., Chugani D.C., Chugani H.T., Donald K.A., Wilmshurst J.M., Shih A. (2016). Autism Spectrum Disorders in Africa: Current Challenges in Identification, Assessment, and Treatment: A Report on the International Child Neurology Association Meeting on ASD in Africa, Ghana, 3–5 April 2014. J. Child. Neurol..

[20] Oneib B., Fajoui Y., El Ghazouani F. (2022). The sociodemographic and clinical profile of children with an autism spectrum disorder in the oriental region of Morocco. Egypt. J. Neurol. Psychiatry Neurosurg..

[21] Sefrioui M.R., Elidrissi I., El Othmani I.S., Derfoufi S., Said A.A.H., Benmoussa A., Derraji S. (2023). Profile of autism spectrum disorders in Morocco: Cross-sectional retrospective study of parents of children with autism. Med. Pharm. Rep..

[22] Fredriks A.M., van Buuren S., Jeurissen S.E., Dekker F.W., Verloove-Vanhorick S.P., Wit J.M. (2004). Height, weight, body mass index and pubertal development references for children of Moroccan origin in The Netherlands. Acta Paediatr..

[23] de Hoog M.L., van Eijsden M., Stronks K., Gemke R.J., Vrijkotte T.G. (2012). Ethnic differences in cardiometabolic risk profile at age 5–6 years: The ABCD study. PLoS ONE.

[24] Hassani G.C. (2015). Facteurs Cliniques et Environnementaux Impliqués Dans la Sévérité du Trouble du Spectre Autistique (à Propos de 50 cas). [Clinical and Environmental Factors Involved in the Severity of Autism Spectrum Disorder (about 50 Cases)]. Ph.D. Thesis.

[25] Elsabbagh M., Divan G., Koh Y.J., Kim Y.S., Kauchali S., Marcín C., Montiel-Nava C., Patel V., Paula C.S., Wang C. (2012). Global prevalence of autism and other pervasive developmental disorders. Autism Res..

[26] Lohman T.G., Roche A.F., Martorell R. (1988). Anthropometric Standardization Reference Manual.

[27] Cole T.J., Bellizzi M.C., Flegal K.M., Dietz W.H. (2000). Establishing a standard definition for child overweight and obesity worldwide: International survey. BMJ.

[28] Mosteller R.D. (1987). Simplified calculation of body-surface area. N. Engl. J. Med..

[29] Box G.E., Cox D.R. (1964). An analysis of transformations. J. R. Stat. Soc. Ser. B (Stat. Methodol.).

[30] Sharma A.K., Metzger D.L., Daymont C., Hadjiyannakis S., Rodd C.J. (2015). LMS tables for waist-circumference and waist-height ratio Z-scores in children aged 5–19 y in NHANES III: Association with cardiometabolic risks. Pediatr. Res..

[31] Cardel M.I., Atkinson M.A., Taveras E.M., Holm J.C., Kelly A.S. (2020). Obesity treatment among adolescents: A review of current evidence and future directions. JAMA Pediatr..

[32] Skinner A.C., Perrin E.M., Moss L.A., Skelton J.A. (2015). Cardiometabolic risks and severity of obesity in children and young adults. N. Engl. J. Med..

[33] Sardinha L.B., Silva A.M., Minderico C.S., Teixeira P.J. (2006). Effect of body surface area calculations on body fat estimates in non-obese and obese subjects. Physiol. Meas..

[34] Leone M., Bui H.T., Kalinova E., Lemoyne J., Gagnon D., Léger L., Larivière G., Allisse M. (2024). Investigation of underlying association between anthropometric and cardiorespiratory fitness markers among overweight and obese adolescents in Canada. Int. J. Environ. Res. Public Health.

[35] Ministère de l’Éducation National, de la Formation Professionnelle, de l’Enseignement Supérieuret de la Recherche Scientifique (2019). Études des Caractéristiques Morphologiques et Sportives de L’enfant Marocain (6–12 ans). Rabat, Maroc. https://jimdo-storage.global.ssl.fastly.net/file/d2c64581-d858-4440-b91e-c2aa12d15ce0/MINISTER%20EDUCATION.

[36] Ghouili H., Ouerghi N., Ben Khalifa W., Boughalmi A., Dridi A., Gmada N., Bouassida A. (2020). First reference curves of waist circumference and waist-to-height ratio for Tunisian children. Arch. Pediatr..

[37] Dempsey J., Dempsey A.G. (2019). Autism Spectrum Disorder Severity, Developmental Delays, and Overweight/Obese Weight Status. J. Pediatr..

[38] Memari A.H., Kordi R., Ziaee V., Mirfazeli F.S., Setoodeh M.S. (2012). Weight status in Iranian children with autism spectrum disorders: Investigation of underweight, overweight and obesity. Res. Autism Spectr. Disord..

[39] Han J.C., Lawlor D.A., Kimm S.Y. (2010). Childhood obesity. Lancet.

[40] Karchynskaya V., Kopcakova J., Klein D., Gába A., Madarasova-Geckova A., van Dijk J.P., de Winter A.F., Reijneveld S.A. (2020). Is BMI a valid indicator of overweight and obesity for adolescents?. Int. J. Environ. Res. Public Health.

[41] Xi B., Zong X., Kelishadi R., Litwin M., Hong Y.M., Poh B.K., Steffen L.M., Galcheva S.V., Herter-Aeberli I., Nawarycz T. (2020). International Waist Circumference Percentile Cutoffs for 722 Central Obesity in Children and Adolescents Aged 6 to 18 Years. J. Clin. Endocrinol. Metab..

[42] Sebbani M., Elbouchti I., Adarmouch L., Amine M. (2013). Prevalence of obesity and overweight among children in primary schools in Marrakech, Morocco. Rev. Epidemiol. Sante Publique.

